# Continuous Sign Language Recognition through a Context-Aware Generative Adversarial Network

**DOI:** 10.3390/s21072437

**Published:** 2021-04-01

**Authors:** Ilias Papastratis, Kosmas Dimitropoulos, Petros Daras

**Affiliations:** Visual Computing Lab at Information Technologies Institute of Centre for Research and Technology Hellas, VCL of CERTH/ITI Hellas, 57001 Thessaloniki, Greece; dimitrop@iti.gr (K.D.); daras@iti.gr (P.D.)

**Keywords:** continuous sign language recognition, sign language translation, generative adversarial networks

## Abstract

Continuous sign language recognition is a weakly supervised task dealing with the identification of continuous sign gestures from video sequences, without any prior knowledge about the temporal boundaries between consecutive signs. Most of the existing methods focus mainly on the extraction of spatio-temporal visual features without exploiting text or contextual information to further improve the recognition accuracy. Moreover, the ability of deep generative models to effectively model data distribution has not been investigated yet in the field of sign language recognition. To this end, a novel approach for context-aware continuous sign language recognition using a generative adversarial network architecture, named as Sign Language Recognition Generative Adversarial Network (SLRGAN), is introduced. The proposed network architecture consists of a generator that recognizes sign language glosses by extracting spatial and temporal features from video sequences, as well as a discriminator that evaluates the quality of the generator’s predictions by modeling text information at the sentence and gloss levels. The paper also investigates the importance of contextual information on sign language conversations for both Deaf-to-Deaf and Deaf-to-hearing communication. Contextual information, in the form of hidden states extracted from the previous sentence, is fed into the bidirectional long short-term memory module of the generator to improve the recognition accuracy of the network. At the final stage, sign language translation is performed by a transformer network, which converts sign language glosses to natural language text. Our proposed method achieved word error rates of 23.4%, 2.1% and 2.26% on the RWTH-Phoenix-Weather-2014 and the Chinese Sign Language (CSL) and Greek Sign Language (GSL) Signer Independent (SI) datasets, respectively.

## 1. Introduction

Sign language (SL) is the primary communication means of hearing-impaired people in their everyday life, and it consists of a well-structured set of grammar rules and vocabulary, similarly to spoken languages. The fundamental element of all sign languages (there are over 300 sign languages used around the world [1]) is the “gloss”, which is composed of a combination of hand shapes, positions, and motion trajectories, orientations of palms and fingers, and facial expressions. Although the development of video-based Sign Language Recognition (SLR) methods for the automated recognition of glosses and the translation of SL to the spoken language is of great importance for the communication of the Deaf community with hearing people (i.e., Deaf-to-hearing communication and vice versa) or among different Deaf communities (i.e., Deaf-to-Deaf communication), it is still considered as a challenging research area. This is mainly because sign languages feature thousands of signs, sometimes differing only by subtle changes in hand motion, shape, or position and involving significant finger overlaps and occlusions [2]. SLR tasks are divided into Isolated Sign Language Recognition (ISLR) [3,4,5] and Continuous Sign Language Recognition (CSLR) [6,7,8]. The CSLR task focuses on recognizing sequences of glosses from videos without predefined annotation boundaries, and it is more challenging compared to ISLR [9], in which the temporal boundaries of glosses in the videos are predefined.

Early SLR methods focused primarily on the classification of simple signs and gestures. They adopted various techniques for extracting handcrafted features [10], e.g., hand and joint trajectories [11] or histograms of oriented gradients [12], while employing traditional sequence-modeling mechanisms, such as Hidden Markov Models (HMMs) [13,14] or Conditional Random Fields (CRFs) [15], for the modeling of the time-evolving information of a sign. On the other hand, recent SLR methods have achieved outstanding performance using deep-learning networks [7,16,17]—in particular, convolutional neural networks (CNNs) and recurrent neural networks (RNNs)—to efficiently extract visual and temporal information from video sequences. Two-dimensional CNNs have already proven their ability to model complex or even heterogeneous data [18], while 3D-CNN or CNN–RNN network architectures have been widely used for spatio-temporal modeling in a variety of applications [19,20,21]. However, and despite the fact that deep generative models have been widely employed in a variety of applications due to their ability to effectively model data distribution, the use of such network architectures in the field of SLR has not been investigated yet. Moreover, existing SLR methods have not attempted to exploit contextual information to further improve the recognition accuracy in sign language conversations.

Generative Adversarial Networks (GANs) are powerful deep generative models that have recently attracted a lot of attention due to their ability to generate novel content, such as high-quality realistic images [22]. GANs consist of two networks: the generator that produces samples and the discriminator, which aims to differentiate the predicted samples from the real ones. In other words, the discriminator tries to determine whether a sample comes from the model distribution or the data distribution [23]. The two networks compete with each other, i.e., a min–max game, aiming to improve the performance of both networks. Due to the fact that GANs can easily learn complicated distributions of data, they have demonstrated impressive results in a variety of tasks, such as video synthesis [24], sign language synthesis [25], and action recognition [26,27].

**Figure 1:**
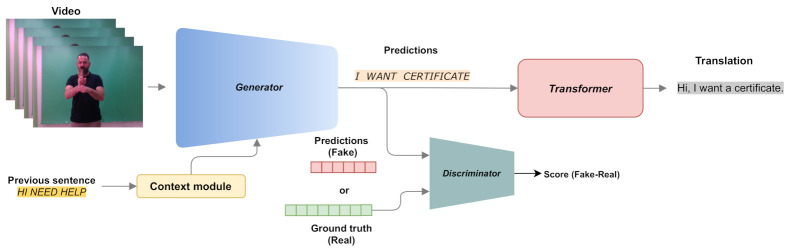
Overview of the proposed framework that performs continuous sign language recognition and sign language translation.

Motivated by the aforementioned analysis, this paper proposes a novel context-aware GAN-based approach for CSLR, namely SLRGAN, which is depicted in Figure 1. More specifically, the proposed network architecture consists of: (i) a video encoder, which plays the role of the generator, aiming to predict the corresponding gloss sequence with respect to the input video, and (ii) a novel discriminator network consisting of a sentence-level and a gloss-level branch to better train the generator and improve gloss prediction. The generator and the discriminator are trained iteratively to compete and learn from each other in a way that improves the CSLR performance of the proposed approach. To take advantage of the context information, i.e., previous sentences from Deaf-to-Deaf or Deaf-to-hearing communication, the use of previous hidden states for the initialization of recurrent neural networks, i.e., the Bidirectional Long Short-Term Memory (BLSTM) layer of the generator, is proposed. At the final stage, the predicted sequence of glosses produced by the generator network is fed into a transformer network, which is based on an encoder–decoder architecture, to perform sign language translation, i.e., turn a gloss sequence into a natural language text.

More specifically, the main contributions of this work are summarized as follows:

- A novel approach for continuous sign language recognition using a generative adversarial network architecture is introduced. The proposed network architecture comprises a generator, which aims to predict the corresponding glosses from a video sequence through a series of a CNN, Temporal Convolution Layers (TCLs), and BLSTM layers, as well as a discriminator, which consists of two branches, i.e., a sentence-level and a gloss-level branch, aiming to distinguish between the ground-truth glosses and the predictions of the generator.
- The importance of leveraging contextual information on sign language conversations is investigated in order to improve the overall CSLR performance. The proposed method uses information from the previous sentence of the dialogue in the form of hidden states to initialize the generator’s BLSTM network in the next sentence for both Deaf-to-Deaf and Deaf-to-hearing communication. Thereby, the previous context of the dialogue is taken into consideration in the next sentence for the recognition of more relative glosses with respect to the conversation topic. The experimental results presented in the paper demonstrate the improvement in sign language recognition accuracy when contextual information is considered.
- The proposed network design was benchmarked on three publicly available datasets and compared against several state-of-the-art CSLR methods to demonstrate its effectiveness. Additional experimental results with a transformer network show the great potential of the proposed method in sign language translation.

The remainder of this paper is organized as follows. In Section 2, related work in SLR is described. The architecture of the proposed CSLR method and the optimization process are described in Section 3 and Section 4, respectively. Finally, the implementation details and the experimental results are discussed in Section 5, while conclusions are drawn in Section 6.

## 2. Related Work

One of the most common methodologies in the literature for tackling the problem of SLR is to employ a feature extraction module to learn visual representations of videos followed by a sequence-modeling module that learns long-term dependencies between the visual representations. Earlier methods were based on HMMs to model sequences, as in [28,29]. Koller et al. [14,30,31] employed hybrid methods using a CNN feature extractor embedded in an HMM to model the gloss transitions, while with the emergence of recurrent neural networks, the same authors [32] extended their work using long short-term memory (LSTM) units for sequence modeling. The aforementioned hybrid methods were trained iteratively using alignments between video frames and glosses provided by the HMM, and they managed to improve the SLR performance with respect to the previous HMM-based methods.

Other approaches employed both recurrent networks and temporal convolutions for sequence modeling, demonstrating the enhanced ability of such combinations to learn complex representations. Moreover, they adopted the Connectionist Temporal Classification (CTC) [33] cost function for training, inspired by the use of the CTC for processing unsegmented input data in other sequence-to-sequence modeling problems, such as speech recognition and handwriting recognition. Cui et al. [34] proposed a 2D-CNN-LSTM network in parallel with stacked temporal 1D convolutions to efficiently capture the temporal boundaries of glosses. In [35], the authors employed a hybrid feature extractor with 2D and 3D convolutional layers, followed by two LSTM networks for sequence modeling at the gloss and sentence levels, respectively, which could be trained end-to-end with CTC loss. In the same direction, many researchers employed different 3D-CNN architectures, inspired by their effectiveness in the human action recognition task [36]. More specifically, in [37], the authors proposed a 3D-CNN network along with a hierarchical attention network for sign language recognition, while in [38], the authors proposed a 3D-CNN along with temporal convolutional and recurrent layers trained with CTC loss. Pu et al. [39] adopted a 3D-ResNet18 as feature extractor, followed by stacked dilated temporal convolutions instead of LSTM units. Zhou et al. [40] adopted the I3D network [36], which was initially proposed for action recognition on top of a recurrent network for temporal modeling. The architecture was trained iteratively with CTC loss and a new pseudo-labeling method. In [41], the authors employed a 3D-Resnet18 with two decoder networks—a CTC decoder and an LSTM decoder, respectively. The whole architecture was jointly trained and aligned with CTC loss and a soft-DTW (Dynamic Time Warping) [42] algorithm, which indicated the alignment path between the video and the glosses. Additionally, the authors in [43] used deep temporal convolution layers for sequence modeling instead of recurrent networks to model the short- and long-term dependencies simultaneously.

On the other hand, other researchers used multiple cues to increase the recognition accuracy, such as optical flow, hand images, and skeleton data. More specifically, in [44], the authors used a two-stream network with 2D-CNNs on top of a BLSTM layer, which trained with RGB (red, green, blue) color images and images of cropped hands, while Koller et al. [8] adopted multi-stream HMMs with synchronization constraints by using powerful CNN-LSTM models in each HMM stream to model RGB, hands, and face data, respectively. In [6], Cui et al. proposed a 2D-CNN followed by temporal convolutions and BSLTM units using both RGB images and optical flow. The whole architecture was optimized iteratively in two stages using CTC loss and cross-entropy loss with pseudo-labels. In [45], the authors proposed a method for exploiting not only RGB data, but also information from multiple cues, such as the pose, hands, and face of the signer, aiming to find correlations between the different cues.

Recently, in [46], the authors proposed an RGB-based CSLR approach using a (2+1)D inception architecture with different receptive fields to extract dynamic spatio-temporal features, while a self-attention network captured the global context. The inception network and self-attention networks were optimized jointly with clip-level feature learning and sequence learning. On the other hand, in [7], a cross-modal approach was proposed for RGB-based CSLR. The extracted video and text representations were aligned into a joint latent space while a jointly trained decoder was employed. However, at the testing phase, only RGB data were used for the task of CSLR. Finally, Cheng et al. [16] proposed a fully convolutional network for online SLR from RGB data and introduced a gloss feature enhancement module to enforce a more accurate sequence alignment.

Over the last few years, GANs have been employed in various research fields, such as video captioning, action recognition, and automatic speech recognition (ASR). In [47], the authors developed a new network architecture for the discriminator to evaluate the video captions based on visual relevance, language fluency, and coherence, while in [26], the authors employed a deep convolutional generative adversarial network for human activity recognition. For speech recognition, in [48], the authors employed a deep speech recognition network trained jointly with a discriminative language model that improves ASR performance. This offers a direction for better utilization of additional text data without the need for a separately trained language model. Other research approaches have leveraged contextual information to improve the accuracy of different network architectures. More specifically, Jiang et al. [49] proposed a hybrid deep learning architecture, where the first unsupervised layer relies on an advanced spatio-temporal Segment Fisher Vector that encodes both visual and contextual features for the automated recognition of mouse behaviors, while Huang et al. [50] introduced a novel generative adversarial privacy framework for designing data-driven context-aware privacy mechanisms. More recently, Zha et al. [51] proposed a Context-Aware Visual Policy network (CAVP) for fine-grained image captioning. During captioning, the CAVP explicitly considers the previous visual attentions as the context and decides whether the context is used for the current word/sentence generation given the current visual attention.

Inspired by the aforementioned research, this work proposes a novel context-aware GAN-based architecture for continuous sign language recognition. Instead of using cross-modal alignment [7] between video and text embeddings, the proposed method employs generative adversarial networks to generate gloss sequences from videos and discriminate between predicted and real gloss sequences (i.e., video embeddings are projected directly to the space of text embeddings), achieving higher sign language recognition performance on three publicly available datasets. In addition, contextual information is employed to improve the accuracy of the proposed network architecture in sign language conversations for both Deaf-to-Deaf and Deaf-to-hearing communication. Unlike other methods, the proposed network architecture extracts spatial and temporal features from video sequences without the need for identifying other visual cues, such as hands [9], skeletal data, facial expressions [3], or optical flow [6].

## 3. Proposed Method

**Figure 2:**
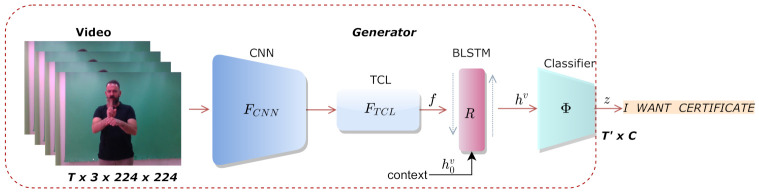
The proposed generator extracts spatio-temporal features from a video and predicts the signed gloss sequences.

The proposed GAN-based network for continuous SLR is shown in Figure 2. The network adopts a video encoder that acts as a generator with the purpose of extracting spatio-temporal features from a video sequence and generating a gloss sequence that best describes the video content. A text-modeling network is employed as a discriminator that aims to model gloss-level and sentence-level text information in order to correctly differentiate between real data and the predictions of the generator. The proposed CSLR approach is trained in an adversarial fashion, where the generator learns to output accurate gloss predictions to increase the error of the discriminator, while the discriminator learns to better differentiate between the real gloss sequences and the generator predictions. The two networks are trained iteratively, competing and learning from each other in a way that improves the overall CLSR performance of the proposed approach. The main components of the proposed architecture are described in detail below.

### 3.1. Generator

The proposed generator, which is illustrated in Figure 2, adopts a 2D-CNN followed by temporal convolution and pooling layers to extract spatio-temporal features from the input frame sequence. The extracted features are then processed through a BLSTM layer to learn long-term dependencies over all time steps. Finally, a softmax layer classifier outputs the predicted glosses. The input frame sequence $x={\left\{x_{\tau }\right\}}_{\tau =1}^{T}$ of length *T* is passed through the 2D-CNN, which is represented by the function $F_{\mathrm{CNN}}$, and extracts the spatial features of the video. In the next processing stage, the 2D-CNN is followed by the TCL module, which is represented by the function $F_{\mathrm{TCL}}$ and consists of stacked 1D convolutions and pooling layers that learn short-term temporal dependencies between frames at each time step τ. The extracted spatio-temporal feature sequence f is represented as follows:

(1) $$f=\{({F_{\mathrm{TCL}}\left(F_{\mathrm{CNN}}\left(x_{\tau }\right)\right))\}}_{\tau =1}^{T},$$

where $f\in R^{T^{\prime }\times D_{F}}$, $D_{F}$ is the output dimension of the feature extractor, and $T^{\prime }=T/\lambda$ is the length of the extracted spatio-temporal sequence, with λ depending on the receptive field of the feature extractor.

In the CSLR task, the signed video is aligned and mapped to a sentence with grammatical rules, meaning that each sign depends on the previous and succeeding signs of the video sequence. BLSTMs have proven successful in learning long-term dependencies that are crucial for correctly recognizing glosses. Moreover, they are capable of encoding both the preceding and succeeding frames, and thus, they provide more powerful video representations than LSTM networks. The BLSTM layer computes the forward and backward hidden sequences and preserves information from both past and future inputs. Using R to represent the BLSTM layer with *K* hidden units, the outputs are computed as:

(2) $$h={\left\{R\left(f_{t}\right)\right\}}_{t=1}^{T^{\prime }},$$

where $h\in R^{T^{\prime }\times K}$ are the concatenated forward and backward hidden state sequences and $f_{t}$ are the extracted spatio-temporal features at time step *t*. Eventually, the concatenated hidden state sequence is passed through a fully connected and softmax layer denoted as Φ, which outputs the gloss label probabilities from a given vocabulary of *C* classes (glosses).

(3) $$z={\left\{\Phi \left(h_{t}\right)\right\}}_{t=1}^{T^{\prime }},$$

where $z\in {\left[0,1\right]}^{T^{\prime }\times C}$ is the output probability distribution of the generator among *C* classes.

### 3.2. Discriminator

**Figure 3:**
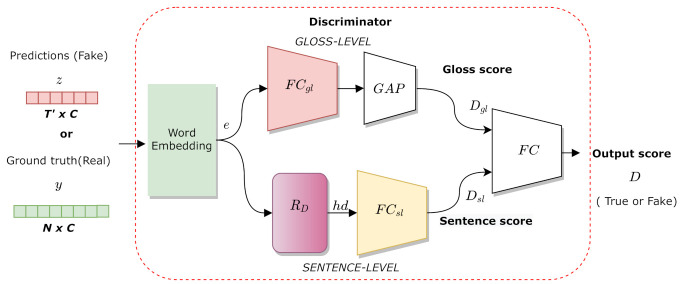
The proposed discriminator aims to distinguish between the ground truth and predicted glosses by modeling text information at both the gloss and sentence levels.

The discriminator is used to distinguish the glosses predicted by the generator from the ground-truth glosses. More precisely, as shown in Figure 3, the discriminator takes either the outputs from the generator or the ground-truth gloss sequence and produces a score between 0 and 1, with 1 representing a real sample and 0 representing a fake sample. The ground-truth gloss sequence of length *K* is represented as a sequence of one-hot vectors $y=(y_{1},y_{2},\cdots ,y_{K})$, while the output probability distribution of the generator is represented as $z=(z_{1},z_{2},\cdots ,z_{T^{\prime }})$. At first, the input sequence is passed through a fully connected layer, i.e., a word embedding that learns a linear projection e. This layer maps the predictions of the generator or the real glosses to a commonly hidden space of a lower dimension. To obtain a score for each gloss, the vector e is passed through a fully connected layer (${FC}_{gl}$), a global average pooling layer (GAP), and a sigmoid function σ. The gloss-level score $D_{gl}$ is calculated as:

(4) $$D_{gl}=\sigma \left(GAP\left({FC}_{gl}\left(e\right)\right)\right).$$

Afterwards, to obtain a sentence-level score for sentence *s*, the vector sequence e is passed through a BLSTM layer ($R_{D}$) to capture long-term relations between glosses. Then, the last hidden state hd of the BLSTM is transformed into a single output through a fully connected layer with a sigmoid activation function (σ). The output score for the entire sentence (sentence-level) is calculated as follows:

(5) $$D_{sl}=\sigma \left({FC}_{sl}\left(hd\right)\right).$$

Finally, the two scores are concatenated and passed through a fully connected layer to obtain the entire score of the discriminator as:

(6) $$D=\sigma (FC\left(\left[D_{gl};D_{sl}\right]\right).$$

### 3.3. Context-Aware SLRGAN

The proposed framework is extended to cover sign language conversations by incorporating the previous context of the dialogue in a way that empowers the network to select relative glosses with respect to the conversation topic. More specifically, the hidden states $h^{v}$ of the BLSTM layer of the generator for the video sequence *v* are initialized with context information from the previous sentence $s_{-1}$ or the previous video sequence $v_{-1}$ for Deaf-to-hearing and Deaf-to-Deaf dialogues, respectively.

Subsequently, the generator outputs the predictions $g=g(v|s_{-1},v_{-1})$ for the video sequence *v* with respect to the previous sentence $s_{-1}$ or the previous video sequence $v_{-1}$. It should be noted that when the generator takes the first sentence of the sign language discussion as input, the initial hidden state is zero, since there is no previous sentence. Finally, to investigate the effect of using relevant information to the sign language conversation and improving CSLR performance, the proposed method was evaluated on Deaf-to-hearing and Deaf-to-Deaf conversations.

#### 3.3.1. Deaf-to-Hearing SLRGAN

**Figure 4:**
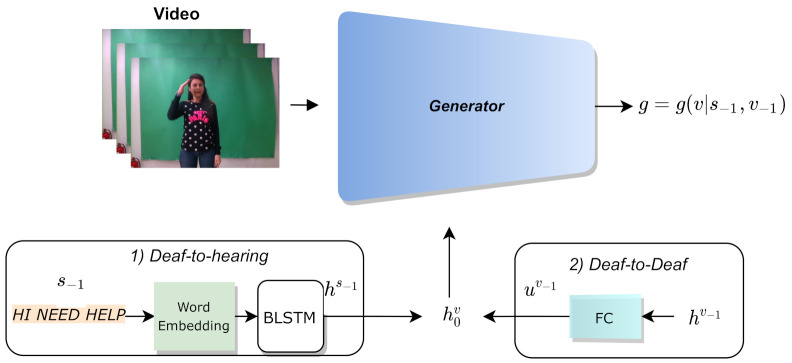
Context modeling on Deaf-to-hearing and Deaf-to-Deaf conversations. In the first case, the previous sentence (text) is passed through a word embedding and a Bidirectional Long Short-Term Memory (BLSTM) layer. In the Deaf-to-Deaf setting, the previous hidden state of the generator is passed through a mapper network. In both cases, the produced hidden state is fed into the BLSTM layer of the generator.

Deaf-to-hearing conversations refer to the communication between a hearing and a Deaf person. A sentence is either directly written by the hearing person or formed by a speech recognition system that captures the speech of the hearing person. This sentence is then fed into the context modeling module of the context-aware SLRGAN to improve CSLR performance on the video with the response of the Deaf person. The spoken text $s_{-1}$ is mapped to the hidden state space by a word embedding and a BLSTM layer, respectively, as depicted in Figure 4. Then, the last hidden state $h_{-1}^{s_{-1}}$ produced by the BLSTM of the context module initializes the hidden state $h_{0}^{v}$ of the BLSTM layer of the generator to improve the predictions of the next video sequence.

#### 3.3.2. Deaf-to-Deaf SLRGAN

Similarly, Deaf-to-Deaf discussions refer to the communication between two Deaf people using sign language. The first person signs one sentence, while the second person responds. The context-aware SLRGAN adopts the context of the discussion to make better predictions of each sentence. In more detail, the hidden states $h^{v-1}$ of the previous video sequence $v_{-1}$ of the discussion are passed through a fully connected layer, as shown in Figure 4. Again, the transformed hidden states $u^{v_{-1}}$ initialize the hidden states $h_{0}^{v}$ of the BLSTM layer of the generator in order to accurately predict the next gloss sequence.

### 3.4. Sign Language Translation

**Figure 5:**
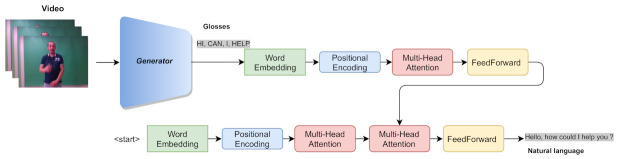
Overall architecture of the sign language translation method. The proposed generator is extended by a transformer network to perform translation of the predicted glosses.

The proposed method is extended by a transformer network [52,53], as depicted in Figure 5, in order to perform sign language translation. The family of the transformer networks follows an encoder–decoder architecture, where both components are composed of *N* identical sub-layers. Each gloss of the input sentence is transformed into a lower-dimensionality vector by an embedding layer before being passed on to the first layer of the encoder in Figure 5. The word embedding is forwarded to a positional encoding layer that adds the information of the relative position of the gloss in the sentence. Then, the embedding is passed through the multi-head attention layer, which employs *H* parallel attention layers, known as heads. This enables the acquisition of multiple representations for a gloss, each one with a different context size, while the model is able to focus on different positions. Then, the outputs of the parallel attention layers are concatenated and passed through the feed-forward layer, which is composed of a fully connected layer. After the multi-head attention and the feed-forward layers, normalization layers and residual connections are employed to ensure stability during training and robustness. The decoder shares a similar structure with the encoder, except for the fact that each sub-layer uses an extra multi-head attention layer, where it takes into consideration the output of the encoder component. At each time step, the decoder outputs a word from the output sentence, which is then fed into the first decoder sub-layer in the next time step, until the symbol indicating the end of the sentence is reached.

## 4. Training

### 4.1. Generator Loss

The CTC loss function is adopted to train the generator when the frame sequence is given. The objective of the CTC loss is to maximize the sum of probabilities of all possible mappings between the input video and the target label sequences. The CTC extends the vocabulary *C* with a blank label ‘‘−", which represents the silence and the transition between two consecutive labels. The extended vocabulary is defined as V=C∪{blank}. Given a frame sequence $x={\left\{x_{\tau }\right\}}_{\tau =1}^{T}$ of length *T*, the proposed generator outputs a gloss probability distribution g with length $T^{\prime }$ to predict the corresponding sequence of target glosses $y={\left\{y_{k}\right\}}_{k=1}^{K}$ of length *K*. The alignment path between the input video and the target gloss sequence is defined as $\pi ={\left\{\pi_{t}\right\}}_{t=1}^{T^{\prime }}$, where the label $\pi_{t}\in V$. Then, the posterior probability of a CTC alignment path π can be defined as:

(7) $$p\left(\pi |x\right)=\prod_{t=1}^{T^{\prime }}g_{\pi_{t},t},$$

where $g_{\pi_{t},t}$ is the emission probability of label $\pi_{t}$ at time step *t*. The alignment path π is mapped to the target sequence y with a many-to-one mapping operation *B* that removes repeated labels and blanks from the given path. Subsequently, an inverse operation $B^{-1}\left(y\right)=\left\{\pi |B\left(\pi \right)=y\right\}$ is used to represent all the possible alignments corresponding to target labels y. The conditional probability of y is defined as the sum of the probabilities of all corresponding paths π:

(8) $$p\left(y|x\right)=\sum_{\pi \in B^{-1}\left(y\right)}p\left(\pi |x\right).$$

The objective function $L_{CTC}$ that guides the training process is derived from the principle of maximum likelihood [54] and is used to optimize the generator. Finally, the CTC loss is formulated as:

(9) $$L_{CTC}=-\log p(y|x).$$

Furthermore, the generator must learn to produce samples that have a low probability of being classified as fake by the discriminator. In addition, the generator minimizes the following loss function in order to output a probability distribution similar to the real data distribution:

(10) $$L_{s}=E_{z∼p_{z}\left(z\right)}\left[\left(1-\log D\left(z\right)\right)\right],$$

where $p_{z}$ is the probability distribution of the predictions *z* of the generator. Finally, the total loss for the generator is defined as:

(11) $$L_{G}=L_{CTC}+L_{s}.$$

### 4.2. Discriminator Loss

The discriminator is trained to maximize the probability of assigning the correct label to both ground-truth glosses and the outputs of the generator. More specifically, the discriminator aims to classify the ground-truth gloss sequence as real, i.e., a score equal to 1, and the output sequence of the generator as fake, i.e., a score equal to 0. The loss function of the discriminator is defined as:

(12) $$L_{D,G}=E_{y∼p_{real}\left(y\right)}\left[\log\left(D\left(y\right)\right)\right]+E_{z∼p_{z}\left(z\right)}[\left(1-\log\left(D\left(G\left(z\right)\right)\right)\right],$$

where $p_{real}$ is the distribution of the ground-truth glosses *y*. The discriminator is trained to minimize the error of assigning the correct label to both distributions. The generator and the discriminator are trained iteratively in each step.

## 5. Experimental Evaluation

In this section, the evaluation metrics and the implementation details are initially described. Subsequently, a series of experiments in three CSLR datasets are performed and discussed. More specifically, the proposed method was evaluated on RWTH-Phoenix-Weather-2014 [11], Chinese Sign Language (CSL) [37], and Greek Sign Language (GSL) [17]. The first two datasets are widely used in CSLR, while the third one is suitable for Deaf-to-Deaf and Deaf-to-hearing sign language recognition. The initial experiments were conducted on the RWTH-Phoenix-Weather-2014 dataset in order to configure the optimal parameters and settings of SLRGAN. Then, the proposed method was evaluated on the three CSLR datasets, and the results are analyzed. Finally, a series of experiments were conducted in order to assess the impact of using contextual information for CSLR and sign language translation.

### 5.1. Datasets and Evaluation Metrics

RWTH-Phoenix-Weather-2014 is the most popular CSLR dataset, and was created from recordings of weather forecasts. Videos were recorded with nine different signers at a frame rate of 25 frames per second. Each video depicts a single German sentence of sign language glosses. The vocabulary size is 1295 and the dataset contains 5672, 540, and 629 videos for training, validation, and testing, respectively.

CSL is a popular CSLR dataset, with a smaller vocabulary compared to RWTH-Phoenix-Weather-2014. The videos were recorded in a predefined laboratory environment with Chinese words that are widely used in daily conversations. The dataset contains 100 predefined sentences performed five times by 50 signers, with 25,000 videos in total. The following signer-independent split (Split 1) [37] of the training and test sets was adopted, with videos from 40 signers used for training and the rest for testing.

GSL is a large-scale Greek CSLR dataset that contains both sign language glosses and spoken language translations. The videos were captured under laboratory conditions at a rate of 30 fps. To increase variability in the videos, the camera position and orientation were slightly altered within subsequent recordings. The dataset consists of five individual and commonly met scenarios in different public services performed by seven different signers. It contains 10,295 videos, with a vocabulary size of 310 unique glosses. Each signer performs signs from pre-defined conversations five consecutive times. In all cases, the simulation considers a Deaf person communicating with a single public service employee. The involved signer performs the sequence of glosses of both agents in the discussion. The dataset has two different evaluation strategies, named GSL Signer Independent (SI) and GSL Signer Dependent (SD) [17]. GSL SI is a signer-independent split, since the recordings of one signer are left out for validation and testing. This split consists of 8821, 588, and 881 videos for the training, validation, and test set, and all sets share the same vocabulary of glosses. On the other hand, GSL SD is a signer-dependent split, where 10% of the recorded sentences are not included in the training set. The sentences in the training set are different from those in the validation and test sets, while the vocabulary used in the validation and test sets is a subset of the vocabulary used in the training set.

GSL and CSL are both balanced datasets, as they contain predefined gloss sequences repeated from each signer. On the other hand, RWTH-Phoenix-2014 is an imbalanced dataset, since it contains unique sentences, and some glosses are signed only once in the entire dataset. It must be noted that for a fair comparison with the other state-of-the-art approaches, data enhancement and resampling were not applied before the training of the network.

For the evaluation of the performance of the proposed method, the word error rate (WER) was selected, since it is the standard metric for the evaluation of CSLR methods’ performance. The WER measures the similarity between the predicted and the ground-truth glosses. More specifically, it calculates the least number of operations (substitutions, deletions, insertions) required to transform the aligned predicted sequence to the ground truth according to the following equation:

(13) $$WER=\frac{S+D+I}{N},$$

where *S* is the total number of substitutions, *D* is the total number of deletions, *I* is the total number of insertions, and *N* is the total number of glosses in the ground-truth sentence.

To evaluate the performance in sign language translation, the Bilingual Evaluation Understudy (BLEU) [55] and METEOR [56] scores were adopted, which are commonly used to measure neural machine translation performance. Concerning the BLEU metric, the BLEU-4 score was adopted. For a fair comparison, the proposed method was compared against state-of-the-art methods that adopt only full-frame (RGB) modality during inference.

### 5.2. Implementation Details

The generator adopted BN-Inception [57] as the 2D-CNN backbone of the network, and was initialized with weights learned with the ImageNet dataset. The kernel and stride sizes of the TCL module were manually tuned to approximately cover the average gloss duration of each dataset. The TCL had two 1D convolutional layers with 1024 filters and two max-pooling layers. For the CSL dataset, the convolutional layers had a kernel size equal to 7, while the pooling layers had kernel and stride sizes equal to 3 and covered the average gloss duration of 58 frames. For the RWTH-Phoenix-Weather-2014 dataset, the convolutional layers of the TCL module had a kernel size equal to 5, while the pooling layers had kernel and stride sizes equal to 2, resulting in a receptive field of 16 frames. Finally, for the GSL dataset, the convolutional kernels and the pooling-layer kernels and strides were set to 5 and 3, respectively. The sequence learning module consisted of two BSLTM layers with 512 hidden units each. The discriminator adopted one BLSTM layer with 128 hidden units and two fully connected layers with 128 filters each. The following data processing techniques were used for all datasets. Each frame of the video was resized to 256×256 and cropped at a random position to a fixed size of 224×224, and up to 20% of the frames in the video were randomly discarded. Videos with more than 300 frames were downsampled due to memory limitations. The generator and the discriminator were trained for 30 epochs. The discriminator was updated after five training steps of the generator in order to stabilize the optimization process. The Adam optimizer with an initial learning rate $\lambda_{0}=10^{-4}$ was used. The transformer implementation was based on the work in [53] and the Open-NMT [58] library. More specifically, the word embeddings had a size of 512 filters and sinusoidal positional encoding, while the feed-forward layers of the transformer contained 2048 hidden units. Furthermore, the encoder and the decoder of the transformer had two layers each. The proposed architecture was implemented in the PyTorch [59] framework. The experiments were conducted on a PC with 64-bit ubuntu 18.04 OS, a Core i7-8700K processor, and 32 gigabytes of RAM. The network was trained on an Nvidia GTX 1080-TI graphics card with the cudNN 7.6.5 and CUDA 10.2 libraries.

### 5.3. Experimental Results

#### 5.3.1. Ablation Study

The initial experiments were conducted using the RWTH-Phoenix-Weather-2014 dataset in order to evaluate the performance of each module of both the generator and discriminator networks. At first, the effectiveness of each module of the generator was investigated. More specifically, Table 1 shows that learning long-term temporal feature dependencies (2D-CNN+BLSTM) is more beneficial than learning short-term temporal feature dependencies (2D-CNN+TCL), with WERs of 27.7% and 29.8% on the test set, respectively. However, the combination of the TCL with BLSTM improved the recognition rate, achieving a WER of 25.0% due to its ability to simultaneously learn short- and long-term feature dependencies. Finally, to verify the importance of modeling both forward and backward hidden sequences, we compared the LSTM with the BLSTM network. As can be seen in Table 1, the use of LSTM (i.e., 2D-CNN+TCL+LSTM network) deteriorated the accuracy of the proposed approach.

Subsequently, further experiments were conducted to find the optimal settings for the discriminator. More specifically, the effectiveness of using the discriminator at the gloss level and sentence level was investigated. From the experimental results in Table 2, it is clear that the GAN-based approach benefits from the adversarial training and outperforms the generator-only approach. At first, the gloss-level discriminator used only the fully connected layers to produce scores for each predicted gloss, and achieved WERs of 23.8% and 23.9% on the validation and test sets, respectively. Subsequently, experiments were conducted with the sentence-level discriminator, which employde a recurrent layer to produce a score for the entire gloss sequence. It was observed that the architecture with the sentence-level discriminator had a slightly worse CSLR performance compared to the gloss-level architecture, with WERs of 23.9% and 24.0% on the validation and test sets, respectively. Finally, the architecture consisting of two branches, i.e., gloss-level and sentence-level branches, was found to improve the overall CSLR performance, with WERs of 23.7% and 23.4% on the validation and test sets, respectively.

#### 5.3.2. Evaluation on the RWTH-Phoenix-Weather-2014 Dataset

In Table 3, the proposed method is compared against several state-of-the-art methods using only RGB data (for fair comparison, methods based on multi-cue information, e.g., [45], are not included in Table 3) on the RWTH-Phoenix-Weather-2014 dataset. SLRGAN achieved a WER of 23.7% on the validation set and 23.4% on the test set. The word error rate was reduced by 0.6% on the test set with respect to [7], which performed cross-modal alignment between the video and text embeddings to model intra-gloss dependencies. This justifies the benefit of improving the output probability distributions by employing a discriminator instead of aligning the two spaces. Furthermore, the proposed method surpassed Re-Sign [32], which adopts an HMM for sequence modeling, as well as the CNN-TEMP-RNN [6] (using only RGB data) architecture, achieving improvements of up to 3.4% and 1%, respectively. In addition, the proposed method performed better than Fully-Conv-Net [16] (improvement of 0.5%), which focuses on gloss features to find better sequence alignments.

#### 5.3.3. Evaluation on the CSL Dataset

The proposed method was also evaluated on the CSL dataset (Split 1) in Table 4. All CSLR methods achieved higher performance on the CSL dataset compared to the RWTH-Phoenix-Weather-2014 dataset because the vocabulary size was smaller (i.e., the CSL dataset had 178 classes, while the RWTH-Phoenix-Weather-2014 dataset had 1232 classes), and there are more training data on the CSL dataset. SLRGAN achieved a WER of 2.1% on the test set, outperforming the SF-NET [35], Fully-Conv-Net [16], and CrossModal [7] methods with absolute WER improvements of 1.7%, 0.9%, and 0.3%, respectively.

#### 5.3.4. Evaluation on the GSL Dataset

The last group of experiments was conducted on the GSL dataset, which contains sign language conversations between signers and enabled us to investigate the use of context from the sign language discussion during recognition.

In Table 5, SLRGAN was evaluated on the GSL dataset and achieved WERs of 2.87% and 2.98% on the validation and test sets of the GSL SI split, respectively. Subsequently, Deaf-to-hearing SLRGAN, which modeled the previous sentence of the conversation, i.e., context, showed a further improvement by reducing the WERs to 2.56% on the validation set and 2.86% on the test set of GSL SI split. Deaf-to-Deaf SLRGAN employde the hidden states of the previous video of the conversation, achieving WERs of 2.72% and 2.26% on the validation and test sets, respectively. It was experimentally shown that the proposed method can produce more accurate predictions when the context of the discussion is used during recognition.

**Figure 6:**
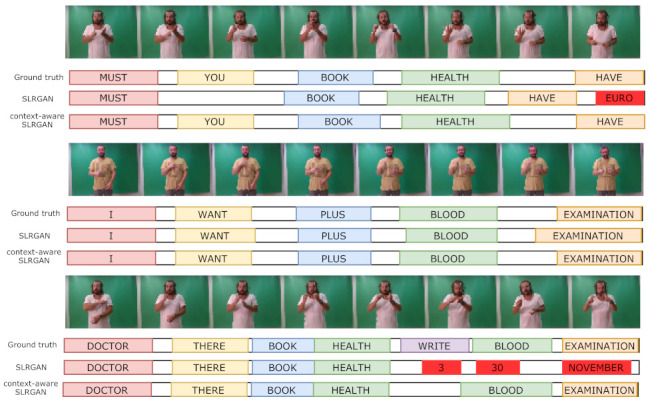
CSLR performance comparison on a sign language conversation. It was observed that the context-aware SLRGAN performs better during recognition of a sign language conversation.

Furthermore, the proposed method was evaluated on the GSL SD split, which is a challenging CSLR task, since the validation and test sets contain different sentences, i.e., unseen sentences, from those in the training set. Nevertheless, as shown in Table 5, SLRGAN outperformed CrossModal [7], with WERs of 36.91% and 37.11% on the validation and test sets, respectively. The results demonstrated the strong generalization capability of SLRGAN in recognizing unseen combinations of glosses. Furthermore, Deaf-to-hearing SLRGAN achieved WERs of 33.75% and 36.68% on the validation and test sets, respectively. Finally, Deaf-to-Deaf SLRGAN achieved a WER of 36.05% on the test set, significantly improving upon all state-of-the-art CSLR approaches. Some qualitative results are shown in Figure 6 from when the proposed method was tested on a sign language discussion between two signers. Overall, it was observed that the context-aware SLRGAN with any setting (Deaf-to-hearing or Deaf-to-Deaf) performs better than SLRGAN and achieves more accurate results by incorporating context information in its predictions. As expected, Deaf-to-Deaf SLRGAN performs slightly better than Deaf-to-hearing SLRGAN, since it employs context from the video instead of text modeling.

#### 5.3.5. Results on Sign Language Translation

In the last set of experiments, the predictions of SLRGAN were fed into the transformer for SLT. In Table 6, SLRGAN and context-aware SLRGANs were used with the transformer for evaluation on the GSL SI and SD datasets. The Deaf-to-hearing SLRGAN+Transformer achieved slightly better performance on the test set, with a BLEU-4 score of 84.91, while the Deaf-to-Deaf SLRGAN+Transformer achieved a BLEU-4 score of 84.96 on sign language conversations compared to SLRGAN+Transformer, which achieved a BLEU-4 score of 84.24. This justifies the importance of context during recognition and translation.

The proposed method was also evaluated on the GSL SD dataset (Table 6). The Deaf-to-hearing and Deaf-to-Deaf SLRGANs with the transformer achieved BLEU-4 scores of 20.33 and 20.26 on the test set, respectively. Moreover, the context-aware SLRGAN+Transformer performed better than SLRGAN+Transformer, which had a BLEU-4 score of 19.34 on the test set, further verifying the need for using context information in sign language recognition and translation. Qualitative sign language recognition and translation results are shown in Table 7. It was observed that the context-aware SLRGAN achieves more accurate predictions than SLRGAN. Furthermore, the use of context improves the quality and fluency of the translations predicted by the transformer.

## 6. Conclusions

In this work, a novel approach that adopted a generative adversarial network for continuous sign language recognition was proposed. Instead of focusing only on visual feature extraction, the proposed approach learns a better representation of the data by taking advantage of adversarial training. To this end, a generator produces gloss predictions from video processing, while a discriminator evaluates the generator’s predictions against the real gloss sequences and learns to differentiate them. By competing against each other, both the generator and the discriminator are improved, ultimately producing more accurate and robust SLR results. Moreover, the proposed method incorporates contextual information that is relevant to the discussion between signers to further improve the CSLR performance for sign language conversations. The experimental results on three large sign language recognition datasets demonstrate the effectiveness of the proposed method. In particular, experiments on the GSL dataset, which contains sign language conversations between signers, demonstrated the improvement of sign language recognition accuracy when contextual information is considered.

Concerning future work directions, we aim to apply the proposed generative adversarial networks for the recognition and translation of different sign languages, as well as to develop a novel mobile application that supports the translation between speech and sign languages to assist people with hearing impairments. In addition, the proposed generator could be adopted in other application fields for the modeling of visual information, such as video captioning and action recognition. Finally, there were several recent research works dealing with the problem of complex and imbalanced data in GAN networks [60,61,62,63,64]. Although the study of this problem is out of the scope of this paper, we consider that future work in this direction can further improve the accuracy of the proposed network architecture.

## Figures and Tables

**Table 1:** SLRGAN performance of the generator with different configurations on the RWTH-Phoenix-Weather-2014 dataset measured with the word error rate (WER).

SLRGAN (Generator Only)	Validation	Test
2D-CNN+TCL (without BLSTM)	30.1	29.8
2D-CNN+BLSTM (without TCL)	27.9	27.7
2D-CNN+TCL+LSTM	26.0	25.8
2D-CNN+TCL+BLSTM	**25.1**	**25.0**

**Table 2:** SLRGAN performance measured with the WER with different discriminator settings on the RWTH-Phoenix-Weather-2014 dataset.

Method	Validation	Test
SLRGAN (generator only)	25.1	25.0
SLRGAN (gloss-level)	23.8	23.9
SLRGAN (sentence-level)	23.9	24.0
**SLRGAN**	**23.7**	**23.4**

**Table 3:** Comparison of the Continuous Sign Language Recognition (CSLR) approaches on the RWTH-Phoenix-Weather-2014 dataset, measured with the WER.

Method	Validation	Test
Staged-Opt [34]	39.4	38.7
CNN-Hybrid [14]	38.3	38.8
Dilated [39]	38.0	37.3
Align-iOpt [41]	37.1	36.7
DenseTCN [43]	35.9	36.5
SF-Net [35]	35.6	34.9
DPD [40]	35.6	34.5
Fully-Inception Networks [46]	31.7	31.3
Re-Sign [32]	27.1	26.8
CNN-TEMP-RNN (RGB) [6]	23.8	24.4
CrossModal [7]	23.9	24.0
Fully-Conv-Net [16]	23.7	23.9
**SLRGAN**	**23.7**	**23.4**

**Table 4:** Evaluation comparison on the Chinese Sign Language (CSL) dataset, measured with the WER.

Method	Test
LS-HAN [37]	17.3
DenseTCN [43]	14.3
CTF [38]	11.2
Align-iOpt [41]	6.1
DPD [40]	4.7
SF-Net [35]	3.8
Fully-Conv-Net [16]	3.0
CrossModal [7]	2.4
**SLRGAN**	**2.1**

**Table 5:** Comparison of Sign Language Recognition (SLR) methods on the Greek Sign Language (GSL) SI and SD datasets.

	GSL SI		GSL SD	
Method	Validation	Test	Validation	Test
CrossModal [7]	3.56	3.52	38.21	41.98
**SLRGAN**	2.87	2.98	36.91	37.11
**Deaf-to-hearing SLRGAN**	**2.56**	2.86	**33.75**	36.68
**Deaf-to-Deaf SLRGAN**	2.72	**2.26**	34.52	**36.05**

**Table 6:** Reported results on sign language translation.

	GSL SI		GSL SD	
	Test		Test	
Method	BLEU-4	METEOR	BLEU-4	METEOR
Ground Truth	85.17	85.89	21.89	28.47
SLRGAN+Transformer	84.24	84.58	19.34	25.90
Deaf-to-hearing SLRGAN+Transformer	84.91	85.26	20.26	26.71
Deaf-to-Deaf SLRGAN+Transformer	84.96	85.48	20.33	26.42

**Table 7:** Qualitative sign language translation results.

**Method**	**Gloss**	**Translation**
**Ground Truth**	HELLO I CAN HELP YOU HOW	Hello, how can I help you?
SLRGAN+Transformer	HELLO I CAN HELP	Hello, can I help?
Deaf-to-hearing SLRGAN+Transformer	HELLO I CAN HELP YOU	Hello, can I help you?
Deaf-to-Deaf SLRGAN+Transformer	HELLO I CAN HELP YOU HOW	Hello, can I help you how?
**Ground Truth**	YOU_GIVE_MY PAPER APPROVAL DOCTOR OWNER OR HOSPITAL	The secretariat will give you the opinion.
SLRGAN+Transformer	ME PAPER APPROVAL DOCTOR	Medical opinion.
Deaf-to-hearing SLRGAN+Transformer	YOU_GIVE_MY PAPER APPROVAL DOCTOR OWNER	Secretariat will give you the opinion.
Deaf-to-Deaf SLRGAN+Transformer	YOU_GIVE_MY PAPER APPROVAL DOCTOR	The secretariat will give you the opinion
**Ground Truth**	YOU HAVE A CERTIFICATE BOSS	You have an employment certificate.
SLRGAN+Transformer	YOU HAVE CERTIFICATE DOCTOR OWNER	You have a national team certificate
Deaf-to-hearing SLRGAN+Transformer	YOU HAVE CERTIFICATE BOSS	You have employer certificate.
Deaf-to-Deaf SLRGAN+Transformer	YOU HAVE CERTIFICATE	You have a certificate.
